# Patterns of Mouse Allergen–Specific IgE and IgG4 in Contemporary Animal Research Environments

**DOI:** 10.1111/cea.70307

**Published:** 2026-04-16

**Authors:** Jennifer Canizales, Susie Schofield, Mohamed H. Shamji, Paul Cullinan, Meinir Jones, Johanna Feary

**Affiliations:** ^1^ National Heart and Lung Institute, Imperial College London London UK; ^2^ NIHR Imperial Biomedical Research Centre London UK; ^3^ London Centre for Work and Health London UK

## Abstract

- Mouse‐sensitised laboratory workers without asthma symptoms have higher mouse‐specific IgG4 than those with symptoms.
- Mouse‐specific IgG4 in workers' serum is associated with inhibiting IgE‐allergen complex binding to B cells.

To the Editor,

Laboratory animal allergy (LAA) results from occupational exposure to allergens found in the hair, dander, urine, and saliva of laboratory animals. It presents as work‐related rhinoconjunctivitis and occupational asthma after a latency period of 2–3 years. Prevalence of rodent‐sensitisation among laboratory animal (LA) workers ranges between 10.4% [1] and 28% [2]. Though exposure is a major risk factor, some individuals develop allergen‐specific IgE without progressing to LAA symptoms and are considered clinically tolerant [3]. High levels of LA exposure are linked to allergic tolerance [2, 4, 5] potentially mediated by tolerance‐associated molecules such as allergen‐specific IgG4, which have been shown to inhibit allergic immune mechanisms [6]. Most LAA studies were conducted before changes in animal housing from open cages to individually ventilated cages (IVCs); this has resulted in lower exposure levels. We examined associations between current exposures, sensitisation, and clinical tolerance in a modern LA workforce.

The SPIRAL study population is described elsewhere [1]. Briefly, 743 LA workers with current occupational exposure to mice completed a self‐reported questionnaire about their occupational exposures and the presence of work‐related asthma, nasal, and ocular symptoms. Participants underwent skin prick testing for common aeroallergens and had blood samples collected. Our analysis included participants who had handled mice in the preceding 12 months and had provided blood samples (*n* = 612). Mouse‐specific IgE was measured using the mouse ‘proteins’ ImmunoCAP (e88) containing a mix of mouse urinary, epithelial, and serum proteins (positive result defined as specific‐IgE level ≥ 0.35 kU/L). Mouse‐specific IgG4 was measured using an established ‘in‐house’ indirect ELISA [7] and standardised against a standard curve of natural human IgG4; a positive result was defined as ≥ 3.0 Arbitary Units (AU)/mL. The mouse extract used for the ELISA was derived from mouse urine. In‐house experiments demonstrated it contained protein bands associated with the main urinary, epithelial, and serum allergens (unpublished data). We performed an exploratory study of inhibition of IgE‐allergen complex binding. LA workers with the top quartile of mouse urine specific IgG4 (*n* = 42) (range: 46.48–410.05 AU/mL) were compared with 44 participants randomly selected from 255 participants who had mouse urine specific IgG4 values below the limit of blank (LOB) of the assay (1.36 AU/mL). The IgE‐facilitated allergen binding (FAB) assay used to determine IgE‐allergen complex binding to CD23 receptors on B cells was performed according to Shamji et al. [8] and Jones et al. [4].

We included 612 individuals (42.8% male, mean age 33.6 years). Median duration of occupational exposure to mice was 5.9 (Interquartile range [IQR]: 2.8–11.8) years. Mouse‐specific IgE and IgG4 were associated with increasing mouse exposure; higher levels of mouse‐specific IgE and IgG4 were seen in workers handling the greatest number of mice, in technicians, and in those with the longest duration of occupational exposure (Table 1).

**TABLE 1:** Association between mouse‐specific IgE and IgG4, and exposures, atopy, symptoms and sensitisation.

Variable	Total population (*N* = 612)			Total population (*N* = 612)		
	*n*	IgE (kU/L)		*n*	IgG4 (AU/mL)	
		GM (95% CI)	*p*		GM (95% CI)	*p*
**Max.** ^a^ **mice handled ever**						
1–50	260	0.07 (0.06, 0.08)	0.049	260	1.95 (1.79, 2.12)	< 0.001
51–100	95	0.08 (0.06, 0.11)		95	2.41 (1.99, 2.93)	
> 100	256	0.10 (0.08, 0.12)		256	4.92 (4.02, 6.01)	
**Job title**						
Technician/other	186	0.11 (0.08, 0.13)	0.001	186	7.00 (5.46, 8.97)	< 0.001
Scientist	426	0.07 (0.07, 0.08)		426	2.04 (1.89, 2.19)	
**Cage type**						
IVC only	170	0.08 (0.06, 0.10)	0.894	170	2.58 (2.19, 3.04)	< 0.001
Mixed cage^b^	344	0.08 (0.07, 0.10)		344	3.50 (3.00, 4.08)	
Open cage only	98	0.08 (0.06, 0.11)		98	2.10 (1.77, 2.50)	
**Duration of exposure (years) in tertiles**						
0.5–3.4	208	0.07 (0.06, 0.08)	0.028	208	2.45 (2.11, 2.85)	< 0.001
3.5–9.5	204	0.08 (0.06, 0.10)		204	2.66 (2.26, 3.13)	
9.6–42	200	0.10 (0.08, 0.13)		200	4.03 (3.28, 4.96)	
**Atopy** ^c^ **(*n* = 574)**						
No	349	0.07 (0.06, 0.08)	< 0.001	349	2.85 (2.49, 3.26)	0.442
Yes	225	0.10 (0.08, 0.13)		225	3.10 (2.63, 3.66)	
**Mouse bite or scratch**						
No	116	0.07 (0.06, 0.09)	0.183	116	2.18 (1.85, 2.56)	0.004
Yes	496	0.09 (0.08, 0.10)		496	3.19 (2.83, 3.59)	
**Work‐related symptoms**						
No	532	0.07 (0.06, 0.07)	< 0.001	532	2.73 (2.46, 3.03)	< 0.001
Yes	80	0.35 (0.20, 0.64)		80	5.08 (3.68, 7.03)	
**Asthma symptoms**						
No	591	0.08 (0.07, 0.08)	< 0.001	591	2.92 (2.63, 3.24)	0.132
Yes	21	1.16 (0.30, 4.46)		21	4.48 (2.43, 8.27)	
**IgE** ^d^ **sensitisation**						
IgE+	58	4.42 (2.82, 6.95)	N/A	58	15.06 (10.63, 21.33)	< 0.001
IgE−	554	0.05 (0.05, 0.06)		554	2.50 (2.27, 2.75)	

Abbreviations: 95% CI, 95% confidence interval; AU/mL Arbitrary units/mL; GM, geometric mean; IVC, individually ventilated cage.

^a^ Data missing for *n* = 1 participant. Based on self‐reported handling of mice. Only includes mice handled not number of mice in the room.

^b^ Mixed cage exposure represents participants who had worked with both IVCs and open cages.

^c^ Skin prick test results missing for *n* = 38 in total population. Atopy defined as a positive saline‐adjusted wheal (measuring ≥ 3 mm) to at least one of three common aeroallergens (cat, grass pollen, *Dermatophagoides pteronyssinus*).

^d^ Sensitisation defined as a mouse‐specific (ImmunoCAP e88) IgE level of ≥ 0.35 kU/L.

Participants who worked with both open and IVC cages (‘mixed cage’) had higher levels of mouse‐specific IgG4 compared to those who had only ever worked with IVCs or open cages; this association was not observed for IgE (Table 1). To account for the healthy worker effect, the same associations were observed in workers with < 3 years exposure to LA's (*n* = 176) (unpublished data).

Fifty‐eight workers were sensitised to mice, with 29 reporting at least one work‐related symptom. Atopy was significantly associated with reporting at least one work‐related symptom (16.4% vs. 10.3%). Linear regression models were used to assess the association between work‐related symptoms and log IgG4 or log IgE; geometric mean ratios (GMRs) and 95% confidence intervals (CI) were reported. After adjusting for confounders (age, sex, atopy, maximum mice handled, and job title), there was no association between IgG4 and any reported work‐related symptoms. Reporting work‐related asthma symptoms was associated with a 68% decrease in the geometric mean of IgG4 compared to workers without (GMR 0.32; 95% CI 0.12, 0.83). For mouse‐specific IgE the inverse was true, reporting work‐related asthma was associated with higher levels of mouse‐specific IgE (GMR 3.53; 95% CI 1.07, 11.66), although confidence intervals were wide.

In the IgE‐FAB assay, workers with the highest levels of mouse‐specific IgG4 (*n* = 42) had greater levels of the inhibition of IgE‐allergen complex binding to B cells compared with IgG4 negative workers (*n* = 44) (72.6% [IQR: 49.1–87.8]; vs. 13.8% [IQR: 4.5–44.9]).

In summary, in the largest multi‐centre study of LA workers to date, we found that proxies of higher exposure, such as job role and the number of mice handled at work, were associated with elevated mouse‐specific IgE and IgG4 antibodies in contemporary animal research facilities. For the first time, we demonstrated that IgG4 levels vary with the type of cage used. Individuals who had worked with both open cages and IVCs (‘mixed cages’) exhibited higher IgG4 levels than those who had worked exclusively with either system. This supports previous work suggesting that variable exposures are associated with increased levels of specific‐IgG4 [9]. Elevated mouse‐specific IgE and IgG4 levels were associated with work‐related symptoms. Among mouse‐sensitised individuals, higher IgG4 levels appeared protective, correlating with fewer reports of work‐related asthma, whereas elevated IgE levels were associated with increased asthma symptoms. Due to wide confidence intervals, these findings should be interpreted cautiously. Nonetheless, the data suggest that in animal research settings, IgG4 may indicate tolerance, whereas IgE may be associated with allergic pathology. Notably, we demonstrated for the first time that mouse‐specific IgG4 may exert a functional inhibitory effect by decreasing IgE–allergen complex binding to B cells. Some inhibition observed in the IgG4‐negative group also suggests IgG4 is not the sole driver of tolerance and other immunomodulatory factors may contribute.

## Author Contributions

J.F., P.C. and M.J. supervised and conceptualised the study. J.C. performed the laboratory experiments, conducted the data analysis, and drafted the manuscript. M.H.S. supervised the IgE‐FAB assay work. S.S. performed the statistical analyses. All authors contributed to critical revision of the manuscript and approved the final version.

## Funding

This research was funded by the National Institute for Health and Care Research (JF) and the Colt Foundation (JC).

## Conflicts of Interest

The authors declare no conflicts of interest.

## Data Availability

The data that support the findings of this study are available on request from the corresponding author. The data are not publicly available due to privacy or ethical restrictions.
